# Centrifugation-induced release of ATP from red blood cells

**DOI:** 10.1371/journal.pone.0203270

**Published:** 2018-09-05

**Authors:** Jordan E. Mancuso, Anjana Jayaraman, William D. Ristenpart

**Affiliations:** Department of Chemical Engineering, University of California Davis, Davis, California, United States of America; Université Claude Bernard Lyon 1, FRANCE

## Abstract

Centrifugation is the primary preparation step for isolating red blood cells (RBCs) from whole blood, including for use in studies focused on transduction of adenosine triphosphate (ATP), an important vasodilatory signaling molecule. Despite the wide use of centrifugation, little work has focused on how the centrifugation itself affects release of ATP from RBCs prior to subsequent experimentation. Here we report that both the centrifugation force and duration have a pronounced impact on the concentration of ATP present in the packed RBCs following centrifugation. Multiple subsequent centrifugations yield extracellular ATP concentrations comparable to the amount released during the initial centrifugation, suggesting this effect is cumulative. Pairwise measurements of hemoglobin and ATP suggest the presence of ATP is primarily due to an increase in centrifugation-induced hemolysis. These results indicate that common centrifugation parameters, within the ranges explored here, can release ATP in quantities comparable to the low end of the range of values measured in typical ATP transduction experiments, potentially complicating experimental interpretation of those results.

## Introduction

ATP release by red blood cells (RBCs) is an important signaling event in the microvasculature that helps to control local blood flow through vasodilation [1–3]. A number of external stimuli have been shown to trigger ATP release from RBCs, including, hypoxia [1, 4–6], hypercapnia [4], and changes in pH [1]. Furthermore, it has been shown that mechanical stresses also induce ATP release [7–15], and that the ATP release is attenuated in diseases such as cystic fibrosis [16], pulmonary hypertension [17], and diabetes [18, 19]. Although several membrane proteins have been implicated in this mechanotransductive release mechanism such as the cystic fibrosis transmembrane conductance regulator CFTR [16], adenylyl cyclase [20], Piezo 1 [13], and pannexin 1 [21, 22], the exact mechanism for ATP release remains unclear. Early studies using filter paper to investigate shear induced release of ATP by RBCs revealed that decreasing pore size leads to an increase in detected ATP concentration [2, 16, 17], suggesting that increased shear triggers increased ATP release. Later work employed continuous flow systems comprised of microbore tubing [7, 8, 14], which yielded a similar correlation between decreased internal tubing diameter and increased ATP concentration. More recent work using microbore tubing has evaluated the effects of storage methods on ATP release from RBCs [23]. The advent of microfluidics afforded the unique opportunity to combine continuous flow detection of ATP with systems on the length scale of the microvasculature. Studies in this regime found that a decrease in the cross-sectional area of the channel led to an increase in detected ATP release [9–11], and began to probe the dynamics of ATP release behavior [12]. Interestingly, recent work by Sikora et al.[24] using vortexing as the mechanism for shear stress has suggested that hemolysis may be the primary release mechanism for shear induced ATP release [25]. However, Sikora et al.’s [24] use of 1-14-day old RBCs may complicate our understanding of this result as stored RBCs are more susceptible to hemolysis than fresh blood cells [26].

In all of the above investigating mechanically-induced ATP release from RBCs, the cells must first be separated from whole blood. For many researchers (c.f. Table 1), this initial preparation involves centrifuging whole blood, aspirating off the plasma and buffy coat, and washing cells through a series of subsequent centrifugation steps. Recent studies probing the effect of centrifugation on RBCs have investigated the effect of these preparation steps on hemolysis and corresponding mechanical properties of the RBCs, demonstrating that centrifugation can affect RBCs under some circumstances. Kiss et al. [27] studied the mechanical properties of human RBCs after centrifugation at 500–1500 *g*, and found that following a 10-min centrifugation RBCs showed no significant reduction in deformability or membrane stability. This observation is important because the deformability of RBC’s has been linked to ATP release [15]. In contrast, Urbina et al. [28] compared the relative damage due to SPLITT fractionation and centrifugation, reporting that after centrifugation at 100–1500 *g* cell viability did not decrease significantly (< 2% hemolysis). They did report, however, that cells experienced significant morphological changes, with unclear impacts on sublethal (non-hemolytic) damage to the cells. Several other studies have reported on the effects of shear stress subhemolytic damage to RBC’s [29–31]. Most recently, Wiegmann et al. [32] analyzed the influence of several standard laboratory procedures on erythrocyte damage. Wiegmann et al.’s [32] results indicate that centrifugation at 900 *g*, from 1 to 4 total centrifugations, increased the free hemoglobin (Hb) in the supernatant and decreased RBC deformability.

**Table 1:** Centrifugation parameters used for separation of RBCs from whole blood prior to subsequent ATP transduction experiments.

Experimental method	Centrifugation Parameters			Experimental ATP Values			Citation
	Force (*g*)	Duration (min)	Repet-itions	Blood type	Hct (%)	[ATP] (μM)	
Filter paper	1000	10	4	Human	10	1–4	Sprague et al., 1996 [2]
	1000	10	4	Human	10	0.5–2	Sprague et al.,1998 [16]
	500	10	4	Human	10	0.24–0.75	Sprague et al., 2001 [17]
Microbore tubing	500	10	4	Rabbit	7	0.4–7	Edwards et al., 2001 [7]
	500	10	4	Rabbit	7	3–10	Sprung et al., 2002 [8]
	500	10	4	Rabbit	7	~0.7–6	Fischer et al., 2003 [14]
	500	10	4	Rabbit	7–10	~6±2	Sprague et al., 2003 [6]
	500	10	4	Human	7	0.19±0.1	Carroll et al., 2006 [19]
	500	10	4	Human	7	0.21±0.07	Subasinghe et al., 2008 [18]
	2000	10	1	Human*	7	~0.05–0.13	Wang et al., 2014 [23]
Microchannels	500	10	4	Rabbit	7	2.5–24	Price et al., 2004 [9]
	500	10	4	Rabbit	7	0.3–0.7	Price et al., 2006 [10]
	500	10	4	Rabbit	7	0.5–7	Moehlenbrock et al., 2006 [11]
	N.R.	5	4	Human	10	0.2–1.6	Wan et al., 2008 [12]
	N.R.	N.R.	N.R.	Human	10	0.6–0.8	Cinar et al., 2015 [13]
Vortexing	500	10	4	Human	10	Rel. Inc.^†^	Sikora et al., 2014 [24]
Viscometer	135	4	3	Human	40	Rel. Inc.	Forsyth et al., 2011 [15]
Hypoxia	500	10	4	Human	20	Rel. Inc.	Sridharan et al., 2010 [21]
	833	5	3	Human	0.1	Rel. Inc.	Bergfeld et al., 1992 [4]
Hypoxia + deformation	500	10	4	Rabbit	7	0.1–1.6	Faris et al., 2008 [5]

ATP concentrations are only included here for healthy untreated controls.

*Indicates the use of RBCs stored for 1–36 days treated using standard protocols.

^†^Indicates researchers who do not report absolute ATP concentrations but who do report a relative increase compared to the control.

Thus, centrifugation clearly causes mechanical stress on RBCs, but to date little thought has been given to the effect of this stress in ATP transduction experiments where the initial preparation step is almost invariably centrifugation. A review of the literature reveals that a wide variety of ATP transduction experiments investigating various driving forces all employed centrifugation (Table 1). Most researchers use centrifugation forces between 500 to 2000 g, with durations of 5 to 10 minutes with three to four repetitions. Notably, these centrifugation parameters are comparable to those examined by Urbina et al. [28] and Wiegmann et al. [32] that demonstrated significant impacts on the RBCs. This observation suggests that RBCs may experience both lysis and morphological changes during the initial centrifugation with a concurrent release in ATP. To date, however, no one has investigated the effect of centrifugation on the release of ATP.

In this work, we systematically investigate the impact of centrifugation on ATP release from human RBCs. We find that both centrifugation force and duration affect the concentration of ATP present in the packed RBCs when centrifugation parameters are in the ranges of 1–10 min and 90–16,000 *g*. We find that subsequent centrifugations of the same sample, at a force of 2,300 *g* and 5 min, again contain amounts of ATP comparable to the initial centrifugation, suggesting the amount of ATP lost throughout preparation is cumulative. Measurements of hemoglobin (Hb) and ATP concentration from the same aliquot of supernatant for multiple centrifugations suggest that ATP present in the packed RBCs is primarily due to hemolysis, rather than mechanotransduction or some other mechanism. Most importantly, we find that the magnitude of ATP in the packed RBCs, using centrifugation parameters within the ranges explored here, is on order of the lower range of ATP concentrations measured in typical investigations of ATP release by human RBCs, suggesting care must be taken when interpreting ATP transduction data in experiments that use centrifugation.

## Methods

Human blood was collected by venipuncture from 11 participants in heparin tubes. All samples were collected under pre-approved Institutional Review Board plans for the study of ATP release from human blood. Participants were recruited via advertisements on social media and provided written consent. To mitigate the potential impact of prolonged storage, all experiments were performed within 6 hours of blood collection.

To determine the effect of centrifugation force on ATP release, whole blood from a participant was centrifuged at room temperature (Eppendorf, Centrifuge 5415 D) a single time using speeds ranging from 1,000 to 13,200 rpm (~90–16,000 g) for 5 minutes. To investigate the effect of centrifugation duration on ATP release, whole blood from a participant was centrifuged once using durations ranging from 1 to 10 minutes at a constant force of 2300 *g*. To investigate the effect of multiple centrifugations, RBCs were centrifuged at 2300 *g* for 5 min. The tube containing each sample was then re-mixed by gently inverting the tube, and centrifuged again at the same force and time. A total of five centrifugations were performed on each sample. Based on the range of centrifugal forces, the shear stress applied to each RBC was approximately between 2.62E-06 Pa and 4.65E-04 Pa.

For all experimental measurements of ATP, the standard Luciferin/Luciferase assay was used. The Luciferin/Luciferase solution was prepared by mixing 300 μL of 1 mg/mL Luciferase extracted from *photinus pyralis* (Sigma) with a mixture of 5.1 mg D-Luciferin (Sigma) in 15 mL of physiological salt solution (PSS, 1.2 mM MgSO4, 2.0 mM CaCl2, 4.7 mM KCl, 11.1 mM dextrose with 1mg/ml bovine serum albumin, 21.0 mM tris(hydroxymethyl)aminomethane, and 140.5 mM NaCl, pH adjusted to 7.4 with 10% HCl solution). Working solutions of 10% RBCs in Luciferin/Luciferase (Sigma Aldrich) and 25% supernatant in Luciferin/Luciferase were prepared from each sample following centrifugation. A higher ratio of supernatant to Luciferin/Luciferase was used as concentrations of ATP in the supernatant tended to be much lower than that in the packed RBCs. Three trial replicates were prepared for each centrifugation time, force, or number and were placed into individual wells on a 96 well plate using 75 μL aliquots. The light signal from the reaction of Luciferin/Luciferase and ATP was captured using 63x magnification on an optical microscope (Leica DMI 3000 B). The signal was amplified using a photomultiplier tube (Hamamatsu, model R1527P) installed in a Photon Technology International 814 system with a high voltage supply for signal amplification. The voltage signal was collected in LabView, and custom scripts were written in MATLAB to convert the voltage signal to photons per second (PPS).

A calibration curve was determined for each day using concentrations of ATP from 0–0.1 μM or 0–1 μM prepared using ATP sodium salt (Sigma Aldrich) in PSS. The calibration curve was used to convert the results from PPS to concentration of ATP. An example calibration curve is shown in S1 Fig.

To test for the possibility of cell rupture (or potentially for transient stress-induced pore formation [33]), we also tested the supernatant following centrifugation for the presence of Hb in solution. Detection of Hb was performed using absorbance spectroscopy (SpectraMax M5) at a wavelength of 414 nm in a standard 96 well plate (optically transparent). Calibration curves were prepared using reagent grade Hb (Sigma-Aldrich). The curves were linear, and a representative calibration curve is shown in S2 Fig.

For the experiments testing centrifugation duration, a total of 8 participants were investigated with three trial replicates each for a total of 24 measurements. For centrifugation force, 9 participants were studied with three trial replicates each for a total of 27 individual measurements. Multiple centrifugations and all tests for Hb used only three participants with three trial replicates each for a total of 9 individual measurements. Due to the relatively small sample sizes and variability between each participant, the data did not necessarily follow a normal distribution. Thus, tests for statistical significance were performed using a nonparametric statistic, the Wilcoxon Rank Sum test (p <0.05) [34]. Outliers for each set were determined as: [ATP] > *q*_3_ + 1.5 * (*q*_3_ − *q*_1_), where *q*_*3*_ is the 75^th^ percentile (third quartile) and *q*_*1*_ is the 25^th^ percentile (first quartile).

## Results & discussion

**Fig 1:**
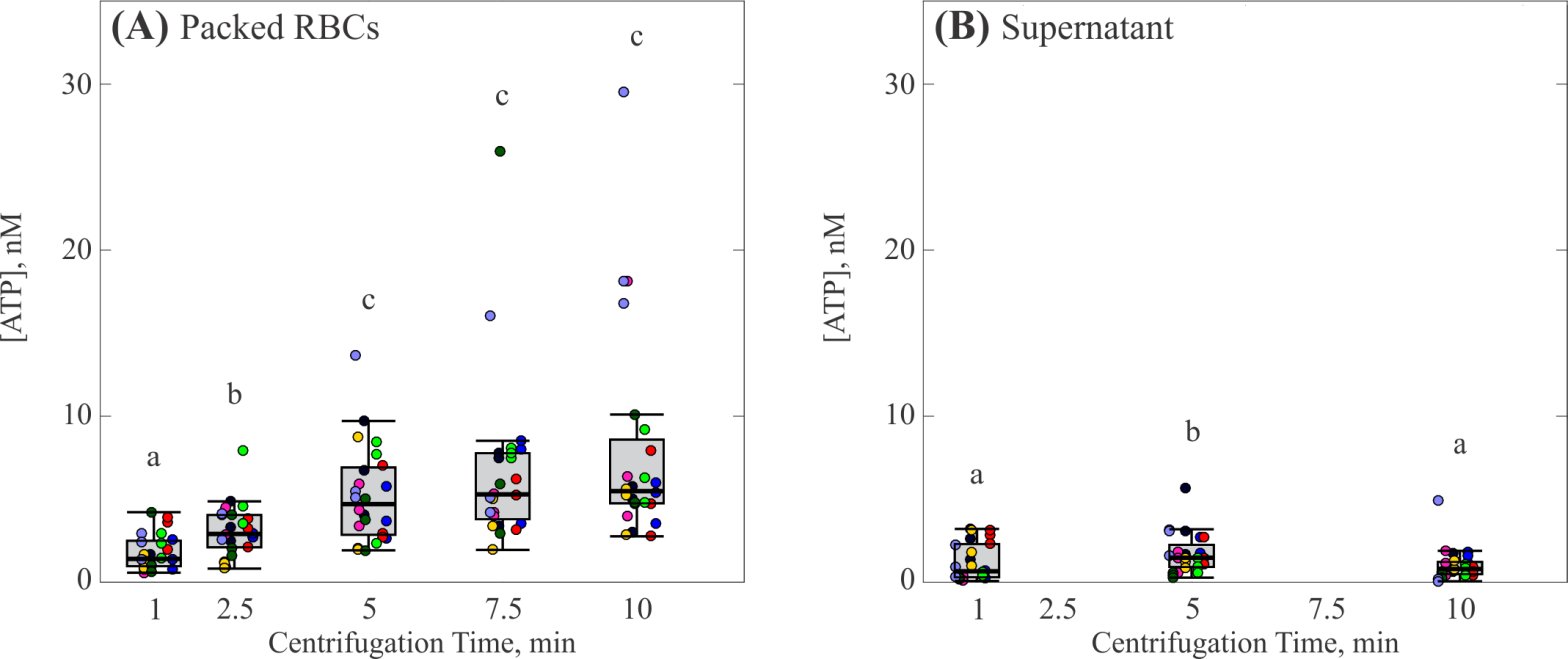
Effect of centrifugation time on ATP release by RBCs. (A) Concentration of ATP in the packed RBCs. (B) Concentration of ATP in the supernatant. Each gray box represents the 25^th^ to 75^th^ percentiles for a sample size of n = 24 measurements from 8 different participants. Black lines inside the boxes represent the median, and whiskers correspond to the maximum and minimum for each position excluding outliers. Colored points represent individual measurements taken from each participant; different colors represent separate participants. Circles above each box represent outliers for each set. Distributions with different letters are significantly different from each other (p < 0.05).

The effect of centrifugation duration is shown in Fig 1, where each box represents the distribution of 24 individual measurements at that specific duration. As centrifugation time increased from 1 min to 5 min, the median concentration of ATP found in the packed RBCs increased from 1.4 nM to 4.7 nM [c.f. Fig 1(A)]. Each median concentration from 1 to 5 min was significantly greater than all lower centrifugation times (p < 0.05). This increase tapered off above a centrifugation time of 5 min, reaching a maximum value of 5.5 nM at 10 min. For each centrifugation time, there was a large range of detected ATP concentrations. The trend for the range standard deviations of detected values followed the trend of the median values, increasing from ±1.1 nM at one min to ±2.9 nM at five min (excluding outliers), after which the increase in the magnitude of the range plateaued. The ATP concentration measured in the supernatant was much lower than in the packed RBCs, with median values from 0.5 nM to 1 nM [c.f. Fig 1(B)]. Notably, the ATP concentration in the supernatant exhibited no correlation with centrifugation time. The standard deviation of detected ATP for each centrifugation time was smaller than that found in the packed RBCs, and became narrower with increasing centrifugation time.

A recurring feature of the measurements was that large spikes were often observed; thus, in order to accurately present this observation, individual measurements have been included in the figures, rather than subsuming them into an average or box plot. The reason for the occurrence of the outliers remains unknown and it can be noted that different samples, even when drawn from the same donor, are necessarily composed of different blood cells. Thus, one potential hypothesis is that particular samples happened to have higher concentrations of RBC’s that were more likely to lyse upon centrifugation. Another possibility is that, despite our best efforts, there were minor differences in centrifugation and handling conditions that somehow affected the blood cells.

**Fig 2:**
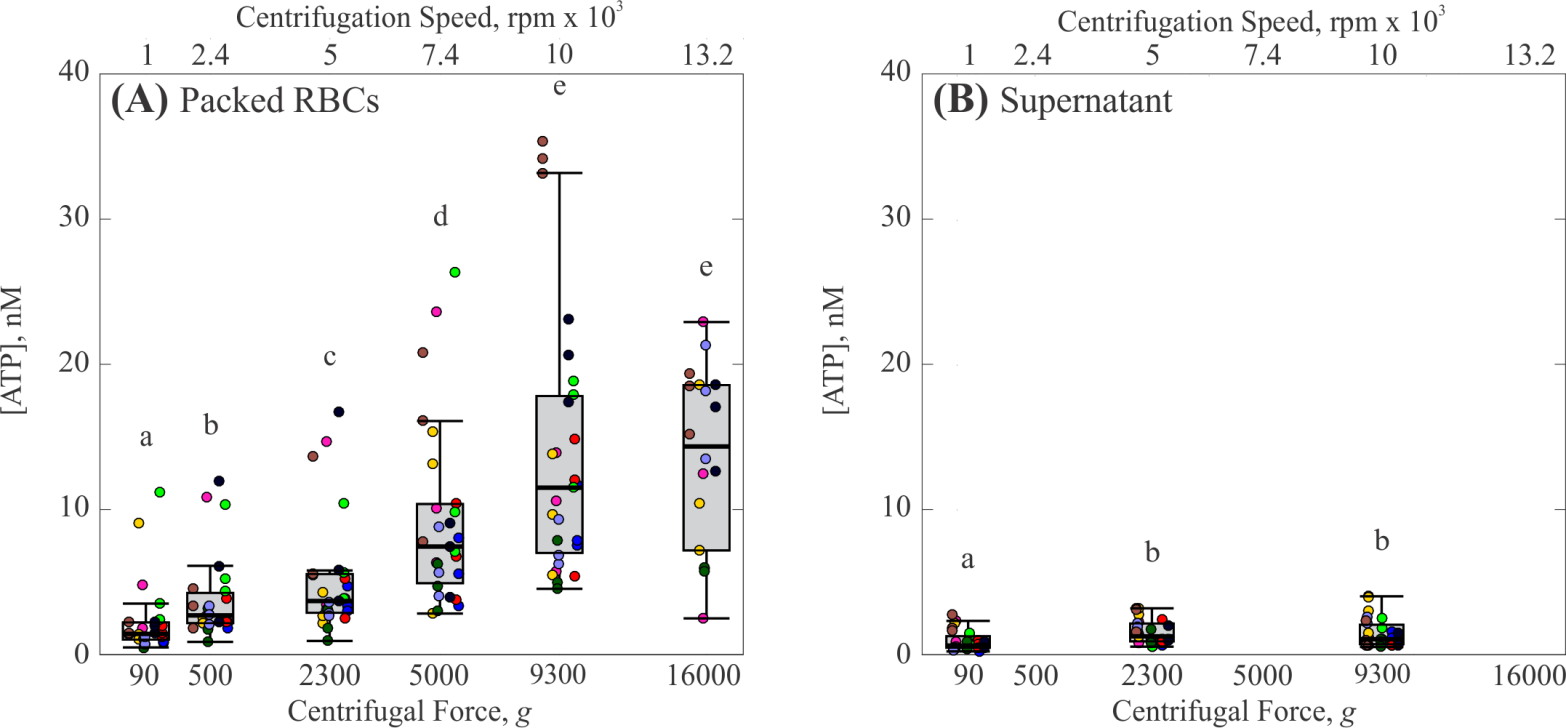
Effect of centrifugation force on ATP release by RBCs. (A) Concentration of ATP in packed RBCs. (B) Concentration of ATP in the supernatant. Each gray box represents the 25^th^ to 75^th^ percentiles for a sample size of n = 27 measurements from 9 different participants, except at 13,200 rpm which represents n = 18 measurements from 8 different participants. Black lines inside the boxes represent the median, and whiskers correspond to the maximum and minimum for each position excluding outliers. Colored points represent individual measurements taken from each participant; different colors represent separate participants. Circles above each box represent outliers for each set. Distributions with different letters are significantly different from each other (p < 0.05).

Centrifugation force yielded a much more pronounced effect on ATP release (Fig 2). As centrifugation force increased from 90 *g* to 16,000 *g*, the median concentration of ATP in the packed RBCs increased by an order of magnitude from 1.4 nM to 14 nM [Fig 2(A)]. Statistical analysis indicates that this increase was monotonic and significant (p < 0.05) for the range from 90 *g* to 9,300 *g*, after which the increase in concentration tapered off. Similar to the results of centrifugation time, the trend for the range of detected ATP concentrations followed that of the median ATP values. The standard deviations of detected ATP increased with increasing centrifugation force, with values of ±2.5 nM at 90 *g* to ±9.0 nM at 9,300 *g* (excluding outliers). Again, concentration of ATP measured in the supernatant was much lower than the packed RBCs, ranging from 0.6 nM to 1 nM, with no dependence on centrifugation force [Fig 2(B)]. The range of detected ATP concentrations in the supernatant for each force was again lower than that found in the packed RBCs. However, in contrast to the results of centrifugation time, the range of detected ATP in the supernatant appeared to increase slightly with increasing centrifugation force.

**Fig 3:**
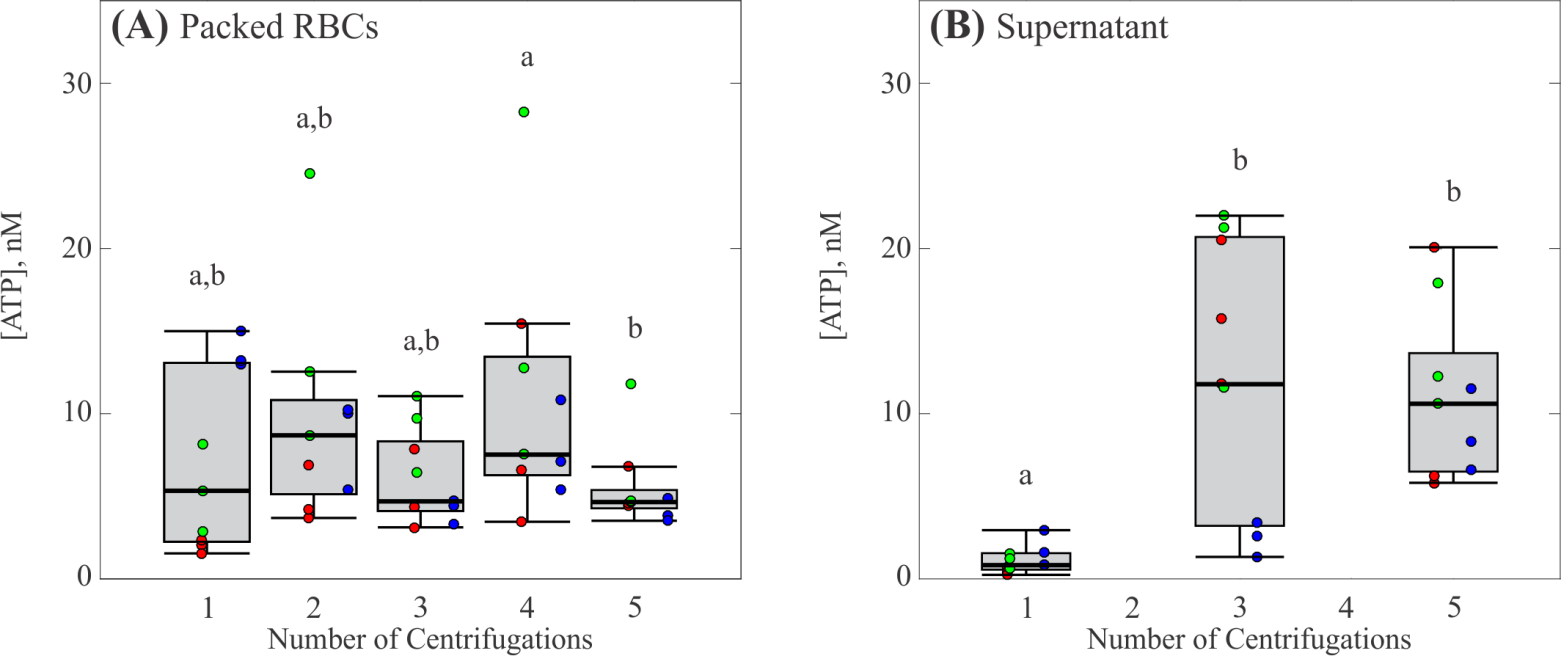
Effect of number of centrifugations on ATP release by RBCs. (A) Concentration of ATP in packed RBCs. (B) Concentration of ATP in the supernatant. Each gray box represents the 25^th^ to 75^th^ percentiles for a sample size of n = 9 measurements from 3 different participants. Black lines inside the boxes represent the median, and whiskers correspond to the maximum and minimum for each position excluding outliers. Colored points represent individual measurements taken from each participant; different colors represent separate participants. Circles above each box represent outliers for each set. Distributions with different letters are significantly different from each other (p < 0.05).

As most researchers preparing isolated RBCs for the study of ATP release perform multiple centrifugations (c.f. Table 1), we also investigated the effect of repeated centrifugations on ATP release by RBCs. The results are shown in Fig 3, where each box represents 3 measurements from 3 participants for a total of 9 individual measurements. The concentration of ATP found in the packed RBCs was relatively unchanged over five total centrifugations with a median concentration of ATP from 5 to 9 nM (Fig 3(A), p < 0.05). Here, the range of detected ATP concentrations tended to decrease slightly with increasing number of centrifugations, with a value of ±7 nM for one centrifugation and a value of ±1.6 nM for 5 total centrifugations. Interestingly, the concentration of ATP in the supernatant increased considerably following the first centrifugation (Fig 3(B), p < 0.05), with median values ranging from 0.8 to 10 nM for one to five centrifugations. The standard deviations increased from ±1.4 to ±2.1 nM between the first and fifth centrifugation. One interpretation of these results is that ATP is released, either due to mechanotransduction or hemolysis, as the cells are pressed together in the bottom of the centrifuge tube. Thus, increases in ATP are not detectable in the supernatant until the tube is gently mixed and re-centrifuged following the first centrifugation.

**Fig 4:**
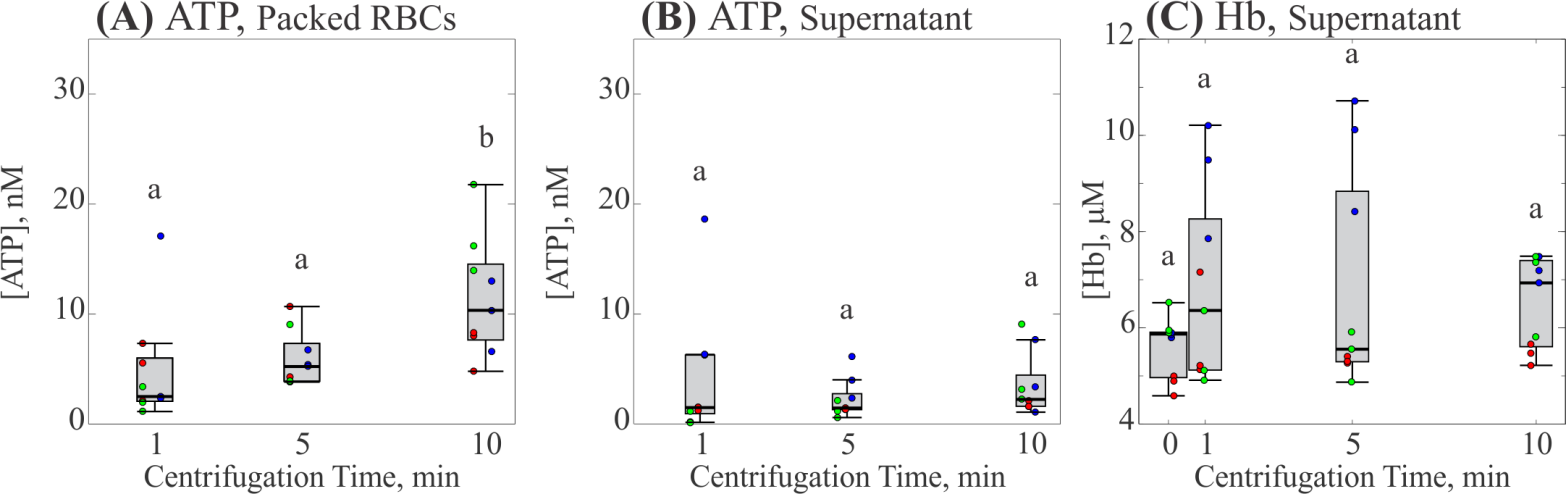
Effect of centrifugation time on ATP and Hb concentrations. (A) Concentration of ATP in packed RBCs. (B) Concentration of ATP in the supernatant. (C) Concentration of Hb in the supernatant. Each gray box represents the 25^th^ to 75^th^ percentiles for a sample size of n = 9 measurements from 3 different participants. Black lines inside the boxes represent the median, and whiskers correspond to the maximum and minimum for each position excluding outliers. Colored points represent individual measurements taken from each participant; different colors represent separate participants. Circles above each box represent outliers for each set. Distributions with different letters are significantly different from each other (p < 0.05).

**Fig 5:**
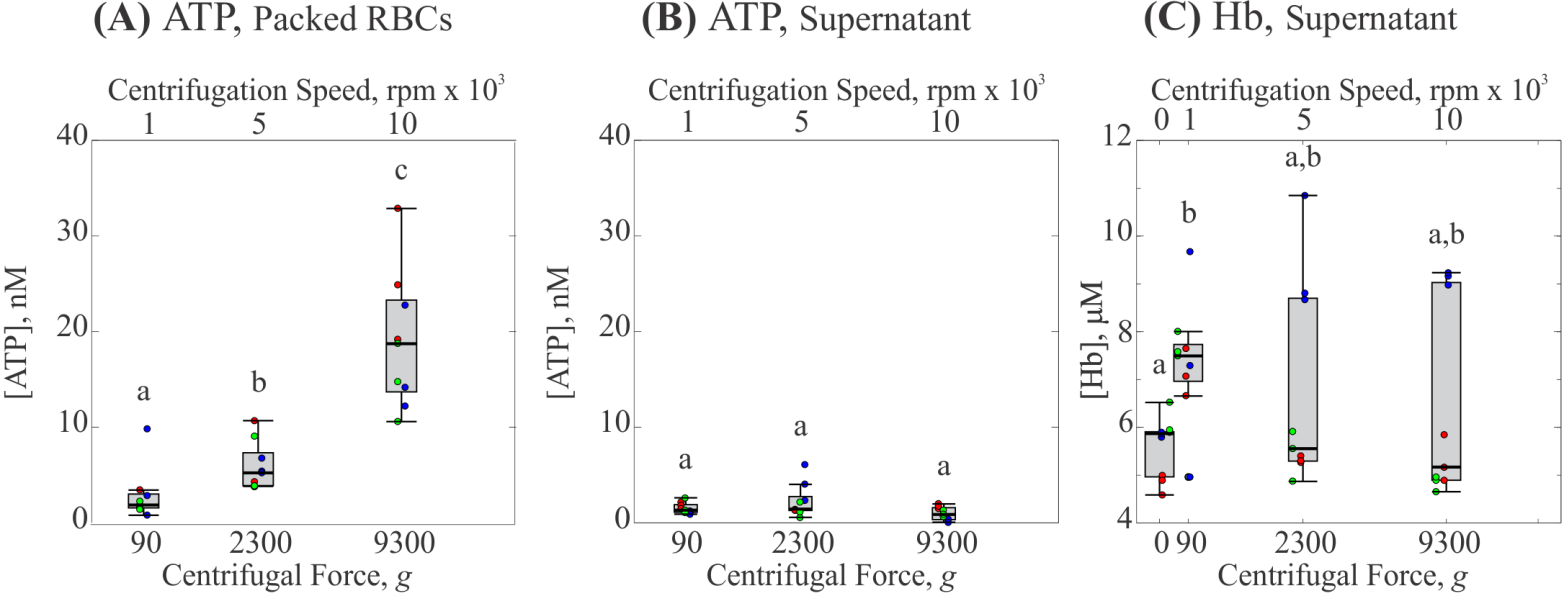
Effect of centrifugation force on ATP and Hb concentrations. (A) Concentration of ATP in packed RBCs. (B) Concentration of ATP in the supernatant. (C) Concentration of Hb in the supernatant. Each gray box represents the 25^th^ to 75^th^ percentiles for a sample size of n = 9 measurements from 3 different participants. Black lines inside the boxes represent the median, and whiskers correspond to the maximum and minimum for each position excluding outliers. Colored points represent individual measurements taken from each participant; different colors represent separate participants. Circles above each box represent outliers for each set. Distributions with different letters are significantly different from each other (p < 0.05).

To determine whether the presence of ATP was due to either hemolysis or mechanotransduction, a completely separate set of experiments was performed in which the same aliquot of supernatant was tested for the presence of both Hb and ATP. Fig 4 and Fig 5 show the effect of centrifugation time and force respectively. In both figures, each box represents 3 measurements from 3 participants for 9 individual measurements. Both experiments conserved the previously observed trends for median ATP concentration in both the packed RBCs and the supernatant. While investigations for hemolysis showed the presence of Hb in the supernatant, it was not significantly greater than the amount detected in a sample allowed to settle naturally (c.f. Fig 4(C) at 0 min and Fig 5(C) at 0 *g*) and was insensitive to increases in either centrifugation time or force.

**Fig 6:**
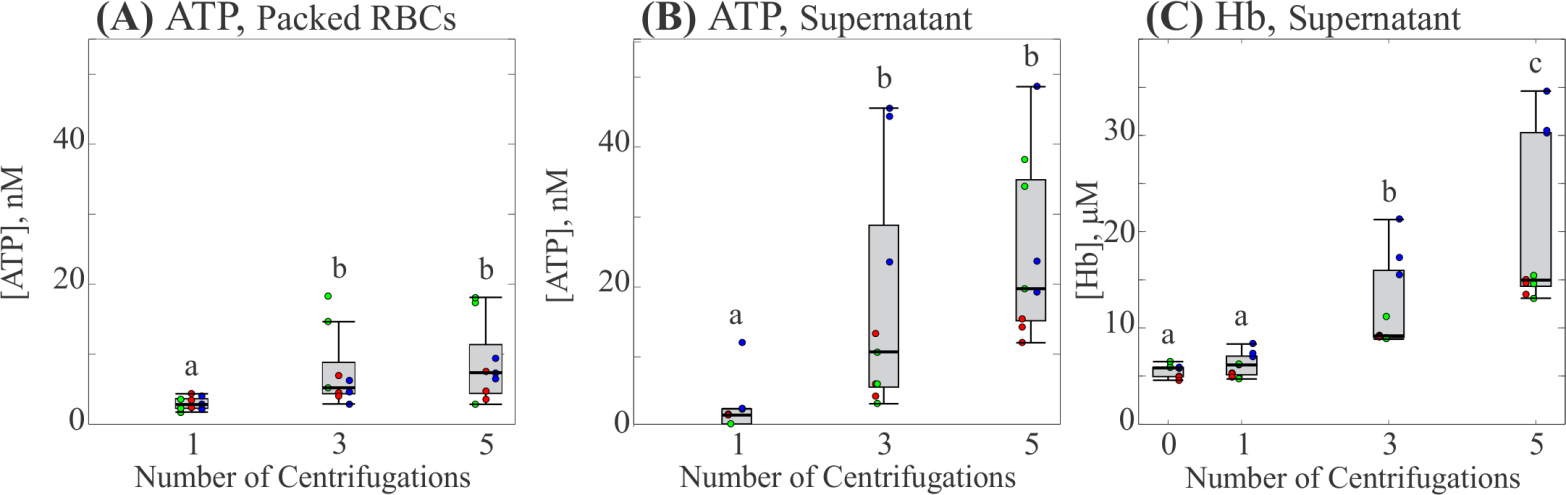
Effect of number of centrifugations on ATP and Hb concentrations. (A) Concentration of ATP in packed RBCs. (B) Concentration of ATP in the supernatant. (C) Concentration of Hb in the supernatant. Each gray box represents the 25^th^ to 75^th^ percentiles for a sample size of n = 9 measurements from 3 different participants. Black lines inside the boxes represent the median, and whiskers correspond to the maximum and minimum for each position excluding outliers. Colored points represent individual measurements taken from each participant; different colors represent separate participants. Circles above each box represent outliers for each set. Distributions with different letters are significantly different from each other (p < 0.05).

Interestingly, when we repeated this pairwise measurement of ATP and Hb over multiple centrifugations we found that both ATP concentration and Hb concentration increased in the supernatant following the initial centrifugation (c.f. Fig 6). However, while ATP concentration appeared to be the same between three and five centrifugations, Hb concentration continued to increase with further centrifugations and was significantly greater than that found in a tube allowed to settle naturally (Fig 6(C) at 0 centrifugations, p < 0.05). This result is consistent with the work of Wiegmann et al. [32] who also observed increases in free Hb in the supernatant with increasing number of centrifugations. As with centrifugation force and time, the earlier trend observed for ATP concentration in the packed RBCs following multiple centrifugations was conserved, albeit with a slight increase following the first centrifugation.

The increase in free Hb in the supernatant following multiple centrifugations indicates an increase in hemolysis. While it is unclear if hemolysis is a major contributor to ATP release in flow systems, our results here strongly suggest that ATP present in the packed RBCs following an initial centrifugation was primarily due to hemolysis, not mechanotransduction. Since we did not detect an increase in either ATP or Hb in the supernatant following an initial centrifugation, one possibility is that hemolysis occurred towards the bottom of the tube as cells were pressed together. Our multiple centrifugation experiments have shown that gentle mixing and re-centrifugation allowed us to detect increases in both ATP and Hb concentration. We hypothesize then, that increasing either the centrifugation force or time leads to an increase in centrifugation induced hemolysis, thereby increasing the concentration of ATP in the packed cells. The concept of hemolysis as a primary ATP release mechanism is consistent with earlier work by Sikora et al. [24]. A key difference here, however, is that Sikora et al. [24] used up to 14 day old RBCs which may increase the probability of hemolysis compared to fresh RBCs [26].

Increasing damage to RBCs caused by increasing shear stress is well known, as indicated by the early couette viscometer work of Blackshear et al. [35] and Leverett et al. [36]. More recent work by Boehning et al. [37] suggested that increased duration of shear increases the probability of hemolysis, even at lower shear stresses, consistent with earlier work by Offeman & Williams [38]. Thus, our hypothesis of increasing hemolysis with increasing centrifugation forces and times is consistent with earlier observations in different shear environments.

Results from multiple centrifugations indicated that hemolysis continued to occur with each subsequent centrifugation, so that overall damage to RBCs throughout the preparation process appears to be cumulative. This result agrees with the previous multiple centrifugation work by Wiegmann et al. [32], who observed[14] increased free Hb in the supernatant as well as decreased cell deformability with each subsequent centrifugation. To determine if the magnitude of hemolysis found in our work is consistent with earlier observations, we calculate an approximate percentage of hemolysis by estimating the number of cells that would need to lyse in order to provide the observed Hb concentrations. Here, we assume an internal Hb content of 27–31 picograms per cell [39], yielding estimates of ~0.08–0.10% hemolysis for one centrifugation and ~0.21–0.24% hemolysis for five total centrifugations. While these percentages are lower than the centrifugation induced hemolysis noted by Urbina et al. [28], it remains unclear whether this lysis is expected to affect the results of further experiments investigating the mechanotransductive release of ATP.

**Fig 7:**
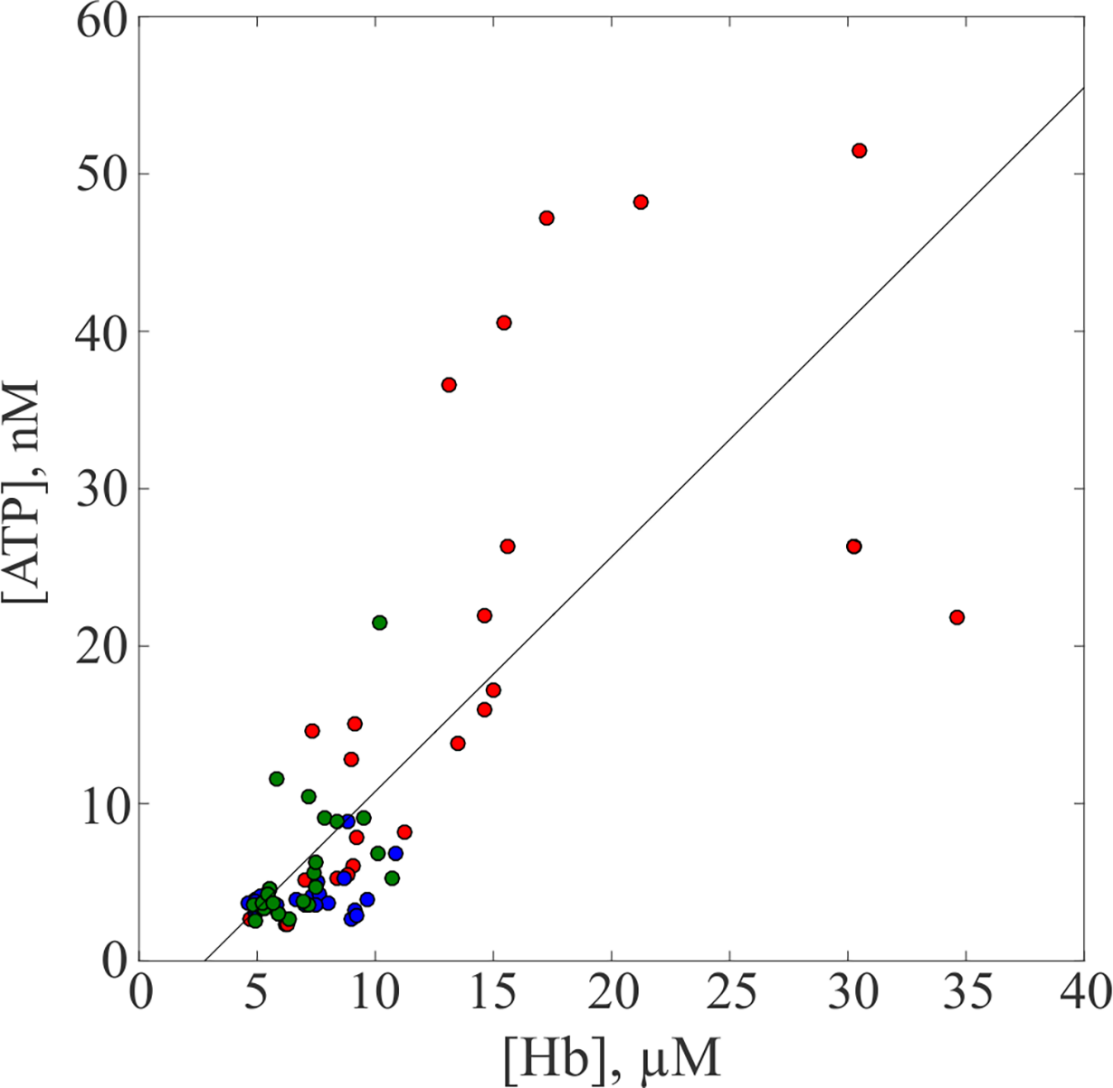
Correlation between Hb concentration and the ATP concentration in the supernatant. The blue, red and green markers represent values from the experiments that studied the effects of force, multiple centrifugations and time respectively. A line of best fit was plotted and the concentrations have a correlation coefficient of 0.773 and p-value of 2.61×10^−17^.

The correlation between the respective concentrations of ATP and Hb in Fig 7 strongly corroborates the original qualitative analysis that the presence of ATP is primarily due to an increase in centrifugation-induced hemolysis.

Typically, to isolate RBCs from other components of whole blood, the cells undergo an initial centrifugation after which the supernatant and buffy coat are aspirated off. Following this, a buffer is added, and the cells are washed and centrifuged three additional times (c.f. Table 1). This amounts to a total of four centrifugations experienced by cells before experiments begin. Results from multiple centrifugations, suggest that over the entire preparation of isolated RBCs, a total of approximately 6–60 nM ATP would be released by hemolysis for centrifugation forces from 90 *g* to 16,000 *g* with a centrifugation time of 5 min.

To understand if ATP release from centrifugation prep is expected to affect subsequent measurements of ATP, we can compare the results reported here to the typical concentrations of ATP released by RBCs in previous studies. Experimentally measured concentrations of ATP from previous work studying ATP release by RBCs are summarized in Table 1. Researchers have reported values from 0.05–4 μM for human RBCs and values from 0.3–24 μM for rabbit RBCs. In comparison, for many of the measurements listed in Table 1, the ATP yielded by centrifugation observed here is negligible. For some experiments with microbore tubing [18, 19, 23], however, the reported ATP concentrations are comparable to those yielded by centrifugation.

## Conclusions

Increasing centrifugation time and force during preparation of isolated RBCs causes an increase in ATP concentration in the packed RBCs. Multiple centrifugations, up to five total, contain similar magnitudes of ATP in the packed RBCs as the initial centrifugation, and supernatant concentrations of both ATP and Hb increase following subsequent centrifugations. The results are consistent with the interpretation that ATP present in the packed RBCs following an initial centrifugation is primarily due to hemolysis, not mechanotransduction, and that subsequent centrifugations increase the number of cells lysed. While for many researchers the concentration of centrifugation-induced ATP might be negligible, this is not always the case. Our results suggest that researchers who anticipate low measurements of ATP release from RBCs should prioritize gentler centrifugation parameters.

## Supporting information

S1 Fig Representative ATP calibration. Points show the average PPS for a given [ATP] in the range of 0–0.1 μM, error bars for each point represent one standard deviation. Since the PPS for a given [ATP] will fluctuate slightly based on the Luciferin/Luciferase solution, a calibration was performed before every experiment.
(TIF)

S2 Fig Representative Hb calibration. Points show the average absorbance for a given [Hb] in the range of 0–25 μM. Error bars for each point representing one standard deviation are too small to resolve here.
(BMP)
