# Supplementary figures and images for: Centrifugation-induced release of ATP from red blood cells
Source: PLoS One. 2018 Sep 5;13(9):e0203270. doi: 10.1371/journal.pone.0203270 (PMC6124747; doi:10.1371/journal.pone.0203270)

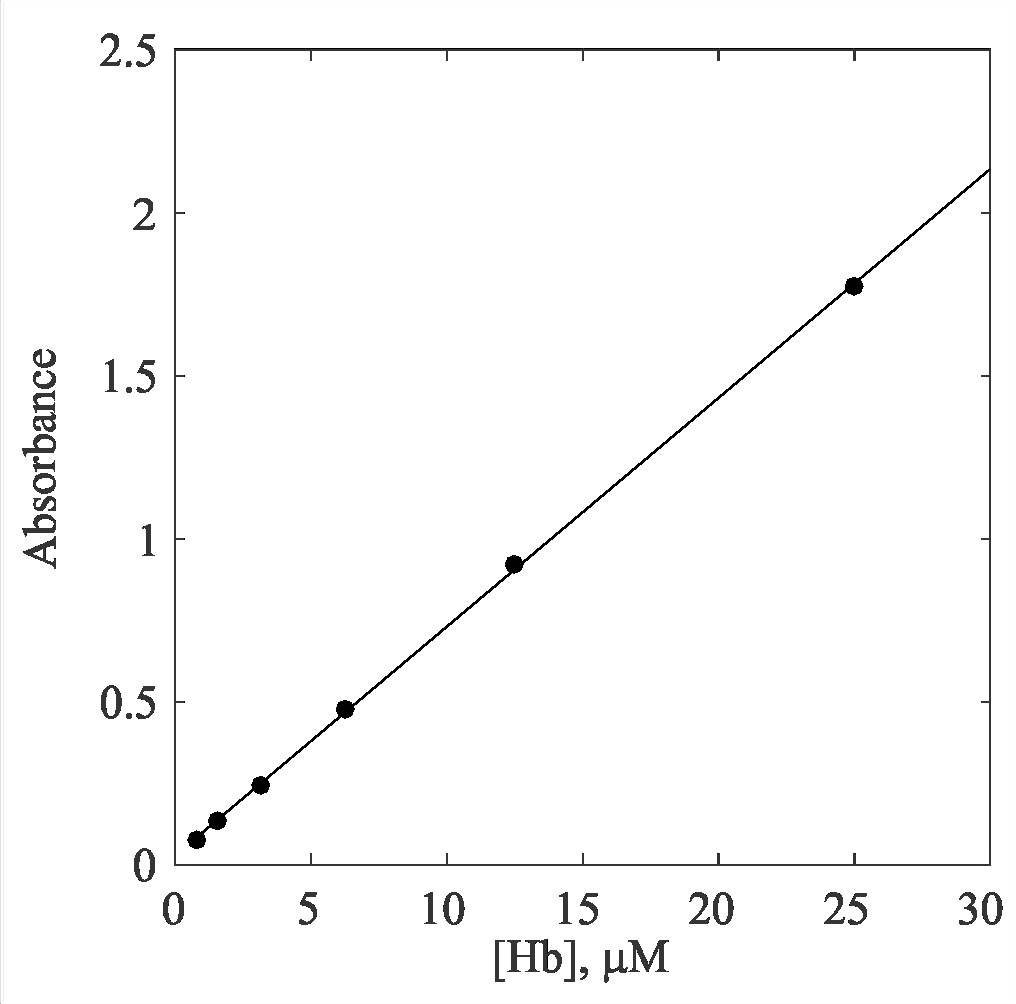

Supplement: S2 Fig — Points show the average absorbance for a given [Hb] in the range of 0–25 μM. Error bars for each point representing one standard deviation are too small to resolve here. (BMP) [file pone.0203270.s002.bmp]
